# Neural basis of shame and guilt experience in women with borderline personality disorder

**DOI:** 10.1007/s00406-020-01132-z

**Published:** 2020-05-07

**Authors:** Martin Göttlich, Anna Lisa Westermair, Frederike Beyer, Marie Luise Bußmann, Ulrich Schweiger, Ulrike M. Krämer

**Affiliations:** 1 Department of Psychiatry and Psychotherapy, University of Lübeck, Lübeck, Germany; 2 Department of Neurology, University of Lübeck, Ratzeburger Allee 160, 23538 Lübeck, Germany; 3 Institute for Psychology II, University of Lübeck, Lübeck, Germany; 4 School of Biological and Chemical Sciences, Queen Mary University of London, London, UK

**Keywords:** Borderline personality disorder, Self-conscious emotions, Shame, Guilt, Amygdala

## Abstract

Borderline personality disorder (BPD) is characterized by instability of affect, emotion dysregulation, and interpersonal dysfunction. Especially shame and guilt, so-called self-conscious emotions, are of central clinical relevance to BPD. However, only few experimental studies have focused on shame or guilt in BPD and none investigated their neurobiological underpinnings. In the present functional magnetic resonance imaging study, we took a scenario-based approach to experimentally induce feelings of shame, guilt, and disgust with neutral scenarios as control condition. We included 19 women with BPD (age 26.4 ± 5.8 years; DSM-IV diagnosed; medicated) and 22 healthy female control subjects (age 26.4 ± 4.6 years; matched for age and verbal IQ). Compared to controls, women with BPD reported more intense feelings when being confronted with affective scenarios, especially higher levels of shame, guilt, and fear. We found increased amygdala reactivity in BPD compared to controls for shame and guilt, but not for disgust scenarios (*p* = 0.05 FWE corrected at the cluster level; *p* < 0.0001 cluster defining threshold). Exploratory analyses showed that this was caused by a diminished habituation in women with BPD relative to control participants. This effect was specific to guilt and shame scenarios as both groups showed amygdala habituation to disgust scenarios. Our work suggests that heightened shame and guilt experience in BPD is not related to increased amygdala activity per se, but rather to decreased habituation to self-conscious emotions. This provides an explanation for the inconsistencies in previous imaging work on amygdala involvement in BPD as well as the typically slow progress in the psychotherapy of dysfunctional self-conscious emotions in this patient group.

**Electronic supplementary material:**

The online version of this article (10.1007/s00406-020-01132-z) contains supplementary material, which is available to authorized users.

## Introduction

Borderline personality disorder (BPD) is defined by a pervasive pattern of instability in interpersonal relationships, self-image, and affect, as well as markedly impulsive behavior [1]. It is associated with a high rate of comorbidity (above 80%), severe psychosocial impairment, intensive use of the health care system, and a high risk of suicide (5–10%) [2, 3]. It is also relatively common with a point prevalence of 1% in the general population, 12% in outpatient, and 22% in inpatient mental health care [4]. The various conceptualizations of this complex disorder all include emotion dysregulation, interpersonal sensitivity, and difficulties with social cognition [5].

As they touch on all these deficits, BPD patients show particularly pronounced levels of the self-conscious emotions shame and guilt [6, 7]. Both are negative affective states following a shortcoming or transgression in an interpersonal situation that is attributed internally [8]. In the case of shame, the attribution is global and stable, resulting in a negative evaluation of the global self (“I did something bad!”). Shame is generally described as feeling small, worthless, and exposed [9, 10] and leads to rumination about the self and personal distress [11]. The corresponding action tendencies are to deny, hide, or escape the shame-inducing situation, e.g. by externalization of blame. This typically leads to intense anger that is expressed destructively [10, 12, 13], thus, shame-proneness is negatively associated with pro-social orientation [8].

In the case of guilt, the transgression is attributed specifically and instable, meaning the focus is on the problematic act and the social other (“I did something bad to them!”) [14–16]. Guilt increases self-reflection and perspective taking, and facilitates reparative behaviors such as confessing, apologizing, and making amends [10, 11]. Therefore, guilt-proneness is associated with pro-social orientation and positive relationship outcomes [8, 17] and has been identified as a component of trait morality [18]. Shame-proneness is associated with a wide range of dysfunctional behavior (such as deliberate self-harm, substance abuse, and risky sexual behavior) and mental health problems (including depression, eating disorder symptoms, and suicidal ideation), whereas the propensity to experience “shame-free” guilt is not [10, 19, 20]. Patients with BPD experience higher levels of state and trait shame than both healthy controls and patients with other axis I or axis II disorders [21–24], whereas guilt correlates negatively with BPD symptoms [25].

Despite the clinical relevance of self-conscious emotions and evidence from questionnaire data speaking for altered shame and guilt experience in BPD, only little experimental work has been conducted on this topic. Gratz et al. [22] found elevated and prolonged feelings of shame in BPD outpatients after a laboratory stressor. This shows that intensified experience of shame in BPD also pertains to experimental situations. Studying shame and guilt in lab experiments poses some challenges. As the attributional style and the self-concept of an individual influences which social emotion is elicited, it varies between subjects [26]. For instance, experiencing an unwanted mishap might trigger embarrassment in some persons, whereas it might be attributed to oneself as a person and thus elicit shame in others. This seems particularly challenging when studying BPD. Failure in achievement situations might be less central to the self-concept of many BPD patients compared to controls [27]. On the other hand, autobiographical memory paradigms might trigger traumatic experiences in persons with BPD more so than in others [28]. In the current study, we took a scenario-based approach. The scenarios all described interpersonal situations, were designed to avoid triggering flashbacks and did not involve any achievement- or job-related situations (see “Methods” for further description).

These scenarios were used to explore the underlying neural correlates of intensified social emotions in BPD. Evoking shame and guilt within the constraints of MRI is even more challenging than in behavioral studies, as the MRI setting heavily constrains actual personal interactions in which social emotions could evolve [26]. The scenario-based paradigm in the current study was designed to experimentally induce feelings of shame, guilt, and disgust and compare them to neutral scenarios. In previous studies using this approach [29, 30], the authors reported activation especially in medial prefrontal areas and superior temporal areas. In addition, experience of guilt elicited insula activation, whereas experience of shame elicited amygdala activity in persons with remitted major depression [31]. A recent review on the neural correlates of shame, embarrassment, and guilt [32] concluded that these social emotions are associated with distinct yet overlapping neural networks, but that results are quite heterogeneous across studies which necessitates further research.

No previous imaging study addressed shame and guilt experience in BPD, but numerous fMRI studies shed light on the neural underpinnings of altered emotional experience and emotion regulation deficits in persons suffering from BPD [33]. Based on the emotion dysregulation theory of BPD [34], many of these studies focused on the amygdala, reporting both increased and decreased activity while processing emotional stimuli [33, 35]. Explanations for these inconsistencies include sample characteristics (small sample size, comorbidities), task methodology (missing emotionally neutral condition), and psychological processes such as habituation or dissociation [33, 35]. Hazlett et al. [36] reported a potentiated amygdala response to repeatedly shown affective pictures in BPD. Patients exhibited normal amygdala activation during novel pictures but relatively increased amygdala activation during repeated pictures compared with HC which supports the notion of altered habituation in BPD.

The complex symptomatology of BPD clearly goes beyond a mere dysfunction of the amygdala. Evidence exists for altered activity in regions implicated in social cognition, empathy, and self-referential processing including the insula, medial PFC, superior temporal sulcus, and precuneus [33]. As mentioned above, several of these regions have also been found in fMRI studies on social emotions, suggesting that dysfunction within this network might be related to elevated levels of shame experience in BPD.

In the present study, we primarily aimed to investigate the neural correlates of elevated shame and guilt experience in women diagnosed with BPD, especially in the above-mentioned networks of social cognition, empathy, and self-referential processing. A secondary goal was to test for correlations between shame/guilt related brain reactivity and disease severity as measured by the borderline symptom inventory (BSI). Motivated by previous experimental evidence of a reduced decline in shame experience over time in BPD patients [22] and theoretical considerations of delayed habituation effects in BPD [34], we explored the temporal evolution of amygdala reactivity during repeated exposure to shame and guilt scenarios.

Addressing the criticisms towards earlier studies reporting partially contradicting findings as discussed above, we included 19 severely affected BPD patients with typical psychiatric comorbidity and 22 healthy controls and included both an emotionally neutral condition and a non-social emotional condition (disgust). We reduced the risk of dissociation by (i) omission of potentially flashback-triggering scenario content, (ii) allowing for familiarization with the study personnel and MRI setting prior to BOLD measurements, and (iii) monitoring of inner tension between runs. Given the heterogeneity of previous imaging results in BPD and on social emotions, we took a whole-brain analysis approach to investigate altered activity for social emotions and specifically for shame experience. To avoid false positives, we controlled the family-wise error rate in our whole-brain analyses. We expected to find enhanced subjective experience of shame in BPD patients and aberrant activations in the amygdala, and brain regions implicated previously in social emotions, namely the medial prefrontal cortex, the anterior insula, precuneus, and inferior frontal gyrus [32].

## Methods

### Participants

All subjects were female, right-handed (self-reported), had German as their native language, were 18–35 years of age, and had normal or corrected to normal vision. Men were excluded in order to gain a sufficiently homogeneous sample. Exclusion criteria for both groups were a history of a central nervous system disease or major head trauma, unwillingness to refrain from alcohol consumption one week prior to each study appointment as well as all MR contraindications (e. g. ferromagnetic or electronic implants).

We recruited 28 women with borderline personality disorder (BPD) at the Department of Psychiatry and Psychotherapy, University of Lübeck, Germany, upon admission to an open ward for inpatient dialectical behavior therapy. All patients were assessed using the German version of the Structured Clinical Interview for DSM-IV axis I and axis II disorders [37]. All women with BPD met criteria for at least one axis I disorder with mood, posttraumatic stress, and eating disorders being the most frequent (Table 1), consistent with other studies [38]. Verbal IQ was measured with a multiple choice vocabulary test (Mehrfachwahl-Wortschatz-Intelligenz test, MWT-B) [39]. The MWT-B is an economic measure of verbal intelligence widely used in psychological research due to its well-established correspondence to more detailed test batteries such as HAWIE-R [40]. Exclusion criteria for the BPD group were a history of schizophrenia, addiction or mental retardation; acute metabolic disturbance (e.g. low potassium), and recent medication with or abuse of benzodiazepines, opioids or alcohol. Other psychotropic medication had to be in steady state at the time of MR measurements and is shown in Supplementary Table 1. From the 28 women with BPD originally recruited to the study, four dropped out for medical reasons (e.g. ingestion of metallic objects), four for personal reasons, and one due to excessive head motion. Their data was not included in the analysis. 13 of the 19 women in the final BPD sample had undergone more than one year of (inpatient or outpatient) psychotherapy prior to recruitment for this study, of which three had undergone more than 5 years.

**Table 1:** Clinical and sociodemographic characteristics of the sample

	Women with BPD (*N* = 19)	Healthy women (*N* = 22)	Test statistic
Age (years)	26.5 (5.8)	26.4 (4.6)	*t*(39) = − 0.68, *p* = 0.946
Verbal IQ	94.8 (6.6)	96.4 (6.5)	*t*(39) = 0.74, *p* = 0.462
BMI (kg/m^2^)	28.7 (10.0)	24.6 (5.0)	*t*(25.5) = − 1.62, *p* = 0.118
Handedness (lateralization quotient)^a^	87.5 (9.9)	76.9 (17.7)	*t*(38) = − 2.53, *p* = 0.030
Secondary education			
None	0.0	4.5^d^	γ = − 0.46, *p* = 0.047
Grundschule (graduation after 4 years)	5.3	0.0	
Hauptschule (8 years)	26.3	4.5	
Realschule (9 years)	42.1	40.9	
Abitur (13 years)	26.3	59.0	
Currently employed	47.4	63.6	*χ*^2^(1) = 1.10, *p* = 0.295
Mental disorders			
Any axis I disorder	100.0		
Mayor depressive episode	63.2		
Social phobia	5.3		
Panic disorder	15.7		
PTSD	36.8		
OCD	10.5		
ADHD	10.2		
Any eating disorder	73.7		
Other personality disorder	10.5		
Questionnaire data			
Borderline symptom inventory (BSI)^b^	33.4 (6.7)	1.0 (1.5)	*Z*^e^ = − 5.53, *p* < 0.001
Shame-proneness^c^	42.7 (8.3)	24.5 (8.5)	*t*(39) = − 6.95, *p* < 0.001
Guilt-proneness^c^	48.7 (5.9)	42.0 (5.6)	*t*(39) = − 3.78, *p* < 0.001
Detachment-proneness^c^	22.3 (4.3)	29.4 (5.4)	*t*(39) = 4.60, *p* < 0.001
External-attribution-proneness^c^	20.1 (5.0)	21.2 (5.0)	*t*(39) = 0.71, *p* = 0.481

*PTSD* posttraumatic stress disorder, *OCD* obsessive–compulsive disorder, *ADHD* attention deficit and hyperactivity disorder

^a^Measured with the Edinburgh Inventory [61]

^b^[45]

^c^Measured with the TOSCA-3 questionnaire [42]

^d^These controls were still attending school. Numbers refer to mean and standard deviation in brackets or percentage values (for secondary education and mental disorders)

^e^Test statistic of the Mann–Whitney *U* test

As controls, 32 healthy women were recruited via local online advertisement and black boards at the local blood donation center, university medical center, public library, and supermarkets. They were matched to age and verbal IQ of the patient group and carefully screened for any current or lifetime axis I or II disorder using the above-mentioned interviews. From the 32 healthy women originally recruited to the study, four dropped out for personal reasons, four for medical reasons (abnormalities detected in structural MR images), and two for excessive head motion. Their data were removed from the analysis, leaving 22 women in the control group.

All participants gave written informed consent after the study protocol had been carefully explained to them. Participants who completed the study received their structural MR images on DVD, 30 €, and a summary of the study’s results in layman terms per email. The study was designed in line with the Declaration of Helsinki and approved by the local ethics committee prior to recruitment (AZ 12-080). Demographic and clinical data of the final study population are presented in Table 1 and Supplementary Table 1. There was no difference between groups regarding age, weight, or verbal IQ. Groups differed with regard to severity of BPD symptoms, handedness, and level of education.

### Experimental paradigm

Participants were presented with short descriptions of fictitious scenarios carrying either shame-, guilt-, disgust-related, or neutral content. The disgust condition served as negative control condition without social aspect. All scenarios were presented in one German sentence written in second-person, present tense (see Supplementary Table 2 for English translations). Scenarios were selected based on theoretical considerations: as their disorder prevents many BPD patients from working, this setting was excluded. Family and relationship settings were omitted to avoid triggering flashbacks. Also, we opted against an individualized approach (e.g. using personal memories) as this would probably result in women with BPD imagining more severe and qualitatively different situations (e.g. sexual abuse) than healthy women, in whom the worst shame-associated memory might be losing one’s head during an oral presentation [26]. Instead, we focused on body shame, as it is of high relevance to the clinical management of BPD and has been linked to both childhood abuse and eating disorder symptoms in adulthood [10]. Scenarios were adapted according to results of pen-and-pencil pilot studies with 32 healthy volunteers and six women with BPD (see Supplementary Table 3 for results of the pilot study).

**Fig. 1:**
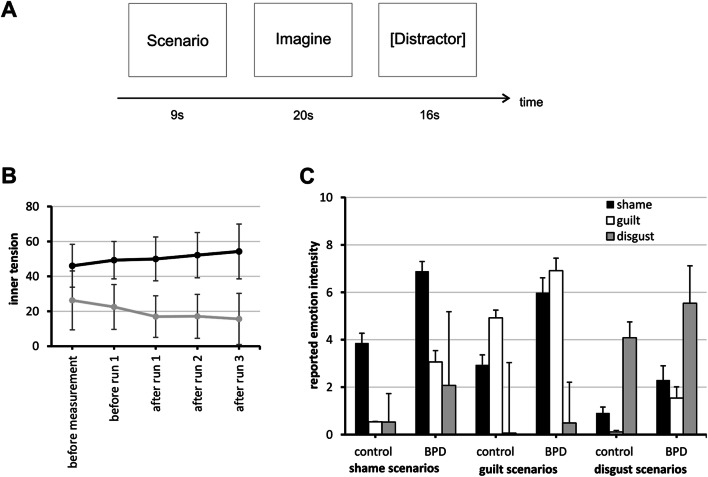
Trial outline and behavioral data. **a** Time-line of one individual trial. **b** Self-reported inner tension over the course of the BOLD measurement in control group (gray line) and BPD group (black line). Error bars indicate one standard deviation. For test statistics see text. **c** Self-reported intensity of shame, guilt and disgust for the three scenario types. Error bars indicate one standard deviation

Scenarios were programmed and presented with Presentation™ (Neurobehavioral Systems, Albany, N.Y., USA), and displayed via VisuaStim Digital™ goggles. In each trial (Fig. 1a), the scenario was presented for 9 s, followed by the German word for “Imagine” for 20 s, followed by a distraction task (pressing a button when the number 3 was presented) for 16 s. In the case of two controls, distraction task data were lost due to technical error. Each run contained the same 12 scenarios (3 scenarios for each condition) in changing, pseudorandomized order; the experiment comprised three runs with a total duration of 30 min. Inner tension was reported orally by participants between runs on a scale from 0 to 100. This form of verbalization is an integral part of the dialectic behavioral psychotherapeutic concept of the open ward we recruited patients from [41]. If patients indicated inner tension level of 100, measurement was to be halted and patients guided through stress resistance skills. This was not necessary in any case. Directly after scanning, participants rated every scenario on 10-point Likert scales regarding vividness of imagination and intensity of their emotional response.

### Study procedures

To level out differences in prior MR experience minimizing emotional responses to the study setting as a potential confounding factor, and to allow for familiarization with the MR setting in order to reduce the risk of dissociation, MR measurements were divided into two appointments, with anatomical and DTI measurements in the first and BOLD measurements of the experimental paradigm in the second. Participants met with the same member of the study team at all appointments, also in order to allow for familiarization. Participants additionally filled out questionnaires: The test of self-conscious affect-3 (TOSCA-3) [42] is a self-report questionnaire using social scenarios to assess proneness to social emotions such as shame and guilt. Participants are asked to rate given behavioral reactions (e.g. “You would think about quitting.”) to the presented scenario (e.g. “You break something at work and then hide it.”) on a five-point scale, based on how likely they would show the reaction themselves. We used the short version with 11 negative scenarios shown to be equivalent to the long version [43]. For the German version, Cronbach’s alpha for the shame-proneness-scale was reported at 0.91, for the guilt-proneness-scale at 0.57 [44].

The borderline symptom inventory (BSI) is a self-report questionnaire consisting of 52 binary items such as impulsivity, instability of identity and relationships, and depersonalization [45]. Affirmative answers are added up to yield a sum-score between 0 and 52. Internal consistency of the BSI is high (reliability as determined with the Kuder-Richardson-20-Formula is 0.92). The cut-off score of 25 [45] differentiates well between BPD patients and healthy controls (sensitivity = 0.54, specificity = 0.80) [46]. An un-validated German translation has been published by [47].

### MRI data acquisition

Structural and functional MRI data were acquired using a 3T Philips INGENIA Omega HP scanner (Philips, Best, The Netherlands). Functional images were acquired using a single-shot gradient echo echo-planar imaging (EPI) sequence sensitive to blood oxygen level-dependent (BOLD) contrast (repetition time TR = 2500 ms; echo time TE = 25 ms; flip angle = 90°; in-plane resolution = 2.5 × 2.5 mm^2^; 47 transversal slices; 2.5 mm slice thickness; 200 × 200 mm^2^ field of view; SENSE factor 2). Additionally, structural images of the whole brain using a T1-weighted 3D TFE sequence (TR = 7.8 ms, TE = 3.6 ms, flip angle 8°, 1 × 1 × 1 mm^3^ resolution) were acquired.

### fMRI preprocessing

Preprocessing was performed using the SPM12 software package (https://www.fil.ion.ucl.ac.uk/spm/). The preprocessing included the following steps: (i) Correction for differences in the image acquisition time between slices; (ii) a six-parameter rigid body spatial transformation to correct for head motion during data acquisition; (iii) co-registration of the structural image to the mean functional image; (iv) gray and white matter segmentation, bias correction, and spatial normalization of the structural image to a standard template (Montreal Neurological Institute, MNI); (v) spatial normalization of the functional images using the normalization parameters estimated in the previous preprocessing step and resampling to 2.5 × 2.5 × 2.5 mm^3^; (vi) spatial smoothing with a 8-mm full width half maximum Gaussian kernel.

Subjects with strong head motion were excluded from the analysis. The six realignment parameters, i.e., three displacements and three elementary rotations with respect to the first image in the EPI series, were used as an estimator for the head motion. The displacements were required to be smaller than 3.0 mm (minimum to maximum) and the individual rotations smaller than 3.0°.

### Statistical analysis

Statistical analyses of behavioral data were carried out using IBM™ Software Package for the Social Sciences (SPSS)™ for windows, version 23.0.0.1.

Functional images were analyzed using a general linear model. On the single-subject level, a design matrix was defined which included one regressor for each of the four read and four imagine conditions. and the distractor task. Brain activity during each trial was modelled using the canonical hemodynamic response function. The design matrix also included the six motion regressors (*x*, *y*, *z*, pitch, roll, yaw) estimated in the head motion correction step during the preprocessing to minimize signal-correlated motion effects. A high-pass filter of 128 s was applied to the data. Classical parameter estimation was performed with a one-lag autoregressive model AR(1) to account for serial correlations in fMRI time series due to aliased biorhythms and un-modelled neuronal activity.

Differences in the processing of shame and guilt scenarios between healthy controls and BPD patients were investigated by a random effects analysis applying voxel-wise two-sample *t*-tests. One-sample *t*-tests were applied to investigate the effect of the experimental manipulation, i.e., brain activation related to the processing of shame and guilt scenarios. To investigate the main effect of shame, guilt, and disgust, we contrasted the imagine phase of those trials with those of the neutral trials. Voxel-wise regression analyses were used to relate changes in regional reactivity with clinical (BSI for BPD patients) and behavioral (TOSCA for patients and controls) data.

Statistical images were assessed for cluster-wise significance using a cluster-defining threshold of *p* = 0.0001. A topological family wise error (FWE) procedure was used to correct for multiple comparisons. The *p* < 0.05 FWE corrected critical cluster size was *k* = 45 voxels. The analysis was performed using SPM12.

## Results

### Questionnaire data

Mean BSI in the patient group was 33.4, which is higher than in other patient groups [45]. Internal consistency of the TOSCA-3 as assessed with Cronbach’s *α* varied between 0.53 (externalization) and 0.92 (shame-proneness). Patients scored higher than controls on the shame-proneness and guilt-proneness subscales, and lower on the subscale detachment. Although groups did not differ in verbal IQ, women with BPD had completed fewer years of school education (see Table 1 for test statistics).

### Behavioral results of experimental paradigm

Mean rates of correct responses in the distraction task were 95.0% (SD 9.6) in the control group and 95.6% (SD 9.0) in the BPD group.

#### Inner tension and dissociation

Self-reported inner tension during measurements did never exceed 80 (*M* = 33.9, SD = 19.9), with mean values ranging between 22.5 (before run 1) and 15.7 (after run 3) in the control group (grand mean = 19.0, SD = 10.7) and 49.3 (before run 1) and 54.3 (after run 3) in the BPD group (grand mean = 50.6, SD = 12.7, see also Fig. 1b). Women with BPD consistently reported higher inner tension than the control group (two-way mixed ANOVA with repeated measurements: *F*(1, 38) = 68.04, *p* < 0.001) There was no main effect for within-subjects factor TIME (Huynh–Feldt-corrected *F*(18.4, 53.43) = 0.54, *p* = 0.569), but a GROUP × TIME interaction (Huynh–Feldt-corrected *F*(1.84, 53.43) = 6.61, *p* = 0.003). Polynomial contrasts revealed a linear decline of inner tension over time in healthy controls [*F*(1, 16) = 4.87, *p* = 0.042] but a linear rise in BPD patients [*F*(1, 13) = 6.61, *p* = 0.023]. In the post measurement questionnaire, all subjects indicated no dissociative symptoms during the measurement.

#### Vividness of imagination

Ratings of vividness of imagination were generally high, with mean ratings between 5.5 (SD = 2.3) and 7.1 (SD = 2.4) out of 10 for healthy control subjects and BPD patients, respectively. Ratings did not differ between groups but between scenario types, as shown by ANOVA with repeated measurements with between-subjects factor GROUP (control, BPD; *F*(1) = 0.01, *p* = 0.911) and within-subjects factor TARGET-EMOTION (shame, guilt, disgust, neutral; Greenhouse–Geisser corrected *F*(2.42, 82.45) = 4.50, *p* = 0.009). Deviation contrasts revealed below-average vividness ratings in guilt scenarios [*F*(1) = 9.31, *p* = 0.004] and above-average ratings in disgust scenarios [*F*(1) = 10.38, *p* = 0.003].

#### Intensity of emotions

Intensity ratings for emotions differed between groups and emotions (Table 2 and Fig. 1c), as shown by three-way mixed ANOVA with within-subjects factors TARGET-EMOTION of the scenario (shame, guilt, disgust, neutral; *F*(3, 90) = 11.34, *p* < 0.001) and type of EMOTION reported (shame, guilt, disgust, fear, surprise, joy, anger, sadness; Huynh–Feldt corrected *F*(4.80, 143.96) = 22.06, *p* < 0.001), and between-subjects factor GROUP (control, BPD; *F*(1, 30) = 7.29, *p* = 0.013). Simple contrasts for TARGET-EMOTION showed higher overall emotion intensity in shame and guilt scenarios as compared to neutral scenarios (all *p* ≤ 0.007). There was a TARGET-EMOTION × EMOTION interaction (Huynh–Feldt-corrected *F*(11.56, 347.89) = 56.09, *p* < 0.001). Pairwise comparisons showed that shame in shame scenarios was rated higher than all other emotions in the same scenario and higher than shame in the other scenarios. The same held true for guilt and disgust (all *p* ≤ 0.006).

**Table 2:** Mean intensity ratings of specific emotions, given separately for groups of participants and types of scenarios

	Shame scenarios		Guilt scenarios		Disgust scenarios		Neutral scenarios	
	Control	BPD	Control	BPD	Control	BPD	Control	BPD
Shame	**3.86 (0.41)**	**6.89 (0.41)**	2.94 (0.42)	5.98 (0.63)	0.91 (0.25)	2.30 (0.60)	1.47 (0.31)	3.02 (0.38)
Guilt	0.54 (.018)	3.06 (0.48)	**4.92 (0.33)**	**6.91 (0.53)**	0.11 (0.06)	1.54 (0.47)	1.25 (0.16)	2.61 (0.27)
Disgust	0.53 (0.21)	2.07 (0.47)	0.06 (0.06)	0.49 (0.25)	**4.08 (0.50)**	**5.54 (0.49)**	0.04 (0.04)	0.19 (0.12)
Anger	2.75 (0.38)	3.63 (0.60)	2.05 (0.48)	3.09 (0.69)	0.89 (0.24)	1.63 (0.50)	0.75 (0.31)	1.02 (0.27)
Fear	0.79 (0.28)	2.67 (0.58)	1.29 (0.33)	2.68 (0.67)	0.33 (0.19)	1.21 (0.46)	0.37 (0.13)	1.49 (0.37)
Sadness	0.92 (0.23)	1.96 (0.65)	1.80 (0.31)	2.79 (0.63)	0.14 (0.06)	0.86 (0.36)	0.37 (0.11)	1.47 (0.34)
Joy	0.41 (0.21)	0.09 (0.06)	0.32 (0.13)	0.23 (0.12)	1.27 (0.31)	1.46 (0.51)	4.26 (0.37)	3.21 (0.44)
Surprise	2.06 (0.47)	1.80 (0.49)	1.62 (0.30)	2.54 (0.55)	2.48 (0.51)	3.49 (0.61)	3.26 (0.53)	2.67 (0.54)

Target emotions are printed in bold. Numbers refer to the mean and standard error (in brackets). See text for ANOVA results

Women with BPD reported higher emotion intensities than healthy women (overall mean difference = 0.96, *p* = 0.013), particularly for shame and guilt scenarios [*F*(3, 90) = 4.37, *p* < 0.001 for the GROUP × TARGET-EMOTION interaction; both *p* ≤ 0.006 in simple contrasts) and higher reported intensities of shame and guilt in BPD women (Huynh–Feldt-corrected *F*(4.80, 143.96) = 7.78, *p* < 0.001 for the GROUP × EMOTION interaction; both *p* ≤ 0.001 in simple contrasts).

### Imaging results

**Fig. 2:**
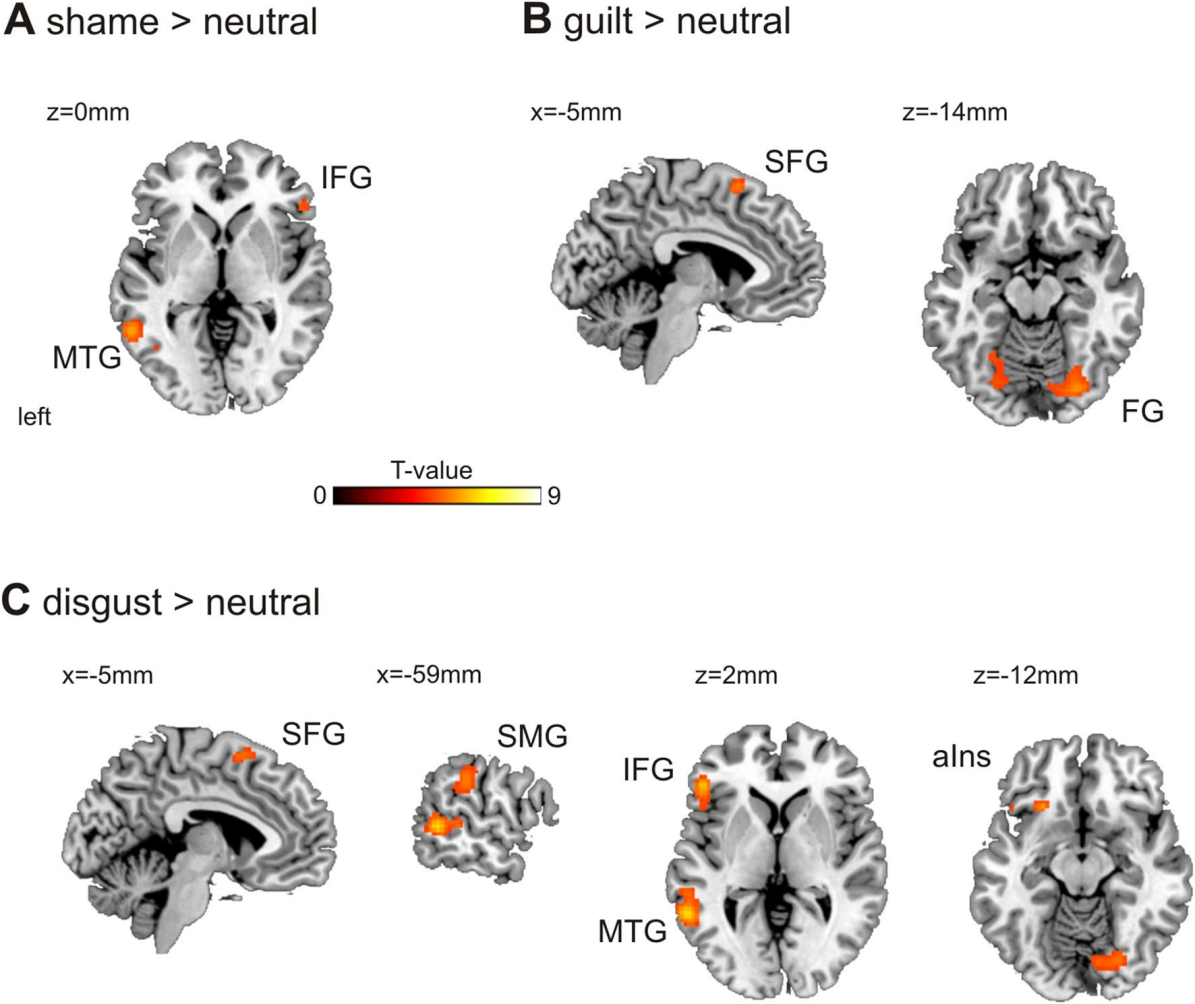
Neuroimaging results. Brain reactivity to social stimuli during the imagine phase of the task. The results are *p* < 0.05 FWE corrected at the cluster level (cluster defining threshold *p* = 0.0001). **a** Stronger brain activation during shame compared to neutral scenarios. **b** Increased activation for guilt vs. neutral scenarios. **c** Stronger activation during disgust vs. neutral scenarios. *aIns* anterior insula, *FG* fusiform gyrus, *IFG* inferior frontal gyrus, *MTG* middle temporal gyrus, *SFG* superior frontal gyrus

During the imagine phase in shame trials, subjects showed stronger activations in the right inferior frontal gyrus and the left middle temporal gyrus (Fig. 2a, Table 3a). Imagining guilt-related situations resulted in stronger activations in the superior frontal gyrus and the occipital fusiform gyrus (Fig. 2b, Table 3b). Disgust-related scenarios led to stronger activations in the left inferior frontal gyrus, the left middle temporal gyrus, the right occipital fusiform gyrus, the left anterior insula, the supramarginal gyrus and the superior frontal gyrus (Fig. 2c, Table 3c). When comparing experimental conditions, we found significantly stronger activations in the inferior/middle temporal gyrus contrasting shame versus guilt trials (Table 3d).

**Table 3:** Imaging results

Brain region	Hem	*p* (FWE) cluster	Cluster size	*p* (FWE) peak	*T* peak	MNI coord. (mm)
A) Main effect: shame > neutral						
Middle temporal gyrus	L	0.003	135	0.021	5.50	− 57, − 50, 0
				0.061	5.09	− 47, − 64, − 5
Inferior frontal gyrus	R	0.026	64	0.030	5.36	48, 30, − 10
				0.240	4.51	53, 28, 2
B) Main effect: guilt > neutral						
Superior frontal gyrus	L/R	0.046	45	0.039	5.31	− 2, 13, 60
Occipital fusiform gyrus	R	0.001	148	0.039	5.31	28, − 80, − 15
				0.350	4.37	10, − 74, − 12
				0.359	4.36	30, − 62, − 22
Occipital fusiform gyrus	L	0.012	80	0.161	4.74	− 24, − 72, − 18
				0.220	4.60	− 24, − 60, − 18
C) Main effect: disgust > neutral						
Inferior frontal gyrus/anterior insula	L	< 0.001	217	< 0.001	8.93	− 50, 28, 8
				0.010	5.82	− 50, 18, 8
				0.360	4.36	− 44, 23, − 10
Middle temporal gyrus	L	< 0.001	197	0.003	6.30	− 57, − 50, 2
				0.153	4.75	− 60, − 37, 2
				0.221	4.59	− 52, − 62, − 5
Occipital fusiform gyrus/cerebellum exterior	R	< 0.001	386	0.003	6.21	26, − 74, − 18
				0.120	4.86	20, − 80, − 8
Anterior insula	L	0.043	47	0.019	5.58	− 24, 23, − 12
Superior frontal gyrus	L/R	0.018	69	0.041	5.29	− 2, 10, 60
Supramarginal gyrus	L	0.013	78	0.082	5.02	− 62, − 30, 35
D) Main effect: shame > guilt						
Middle/inferior temporal gyrus	L	0.014	82	0.072	5.03	− 54, − 52, − 10
				0.102	4.89	− 52, − 60, − 8
E) BPD > HC: guilt + shame > disgust						
Amygdala	R	0.047	46	0.015	5.65	20, 0, − 20

Significant activations for shame (**A**), guilt (**B**) and disgust (**C**) vs. neutral scenarios. **D** Main effect shame > guilt. **E** Significant interaction between the group factor and emotional content, i.e., shame and guilt scenarios on the one hand and disgust scenarios on the other hand. The table shows three local maxima more than 8.0 mm apart

In the next step, we examined group differences in the contrasts shame vs. neutral, guilt vs. neutral and disgust vs. neutral. Uncorrected data for between-group effects (guilt vs. neutral and disgust vs. neutral) applying a voxel level threshold of *p* < 0.001 and a minimal cluster size of *k* = 10 voxels are listed in Supplementary Table 4. At this uncorrected level, guilt scenarios resulted in a higher reactivity in BPD in the anterior insula, the angular gyrus, the precentral gyrus and the entorhinal area. No between-group effect at this significance level was found for the contrast shame vs. neutral. Brain activity evoked by emotional stimuli did not correlate with questionnaire data (BSI for BPD patients and TOSCA for patients and controls).

**Fig. 3:**
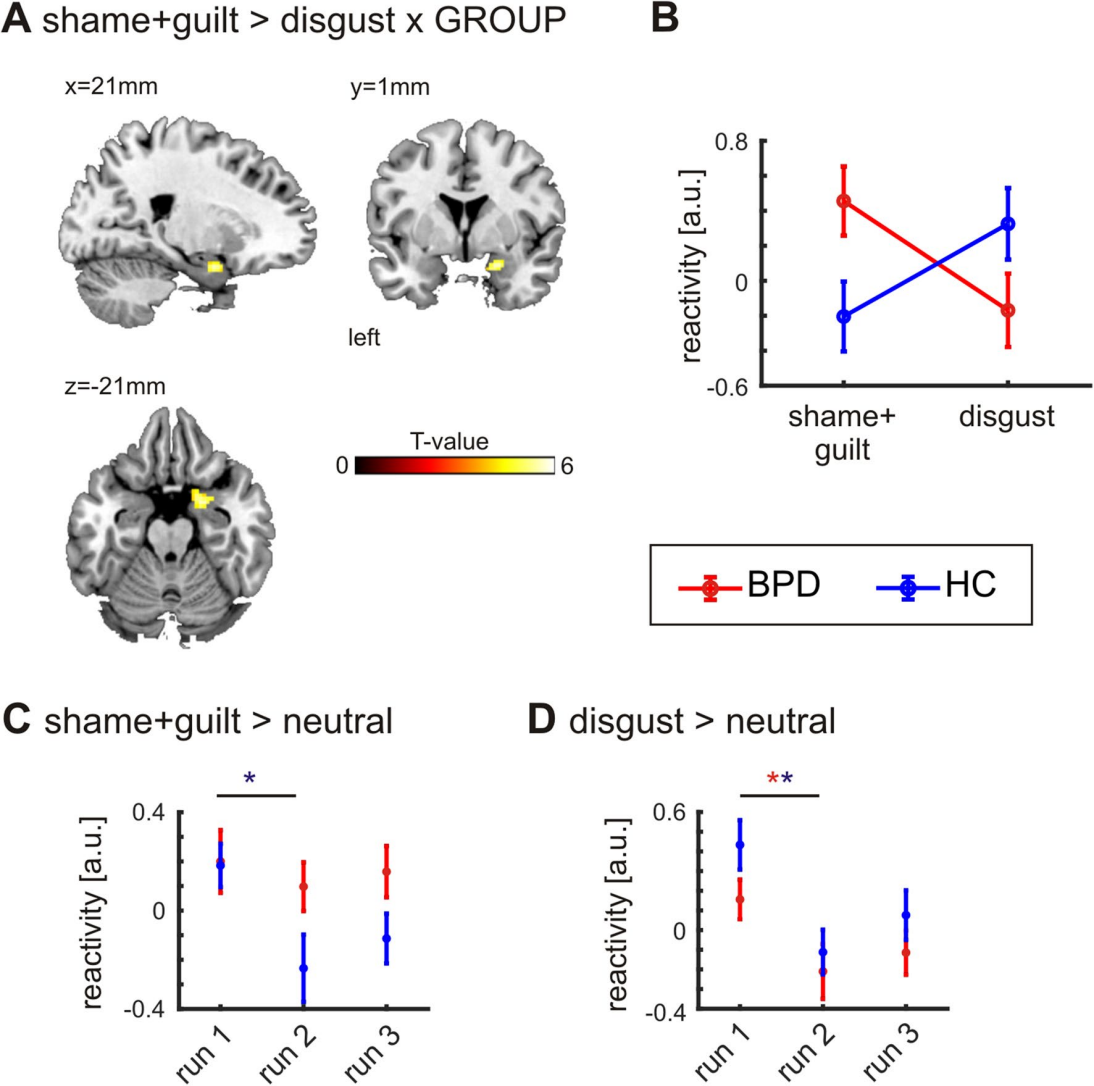
Group differences in amygdala activation. **a** Significant interaction between emotional content and group factor in the right amygdala. **b** The control group shows amygdala activity for disgust scenarios but no reactivity to shame and guilt scenarios, the BPD group shows the opposite effect. **c** The contrast shame + guilt vs. neutral for each run separately, showing a habituation effect in HC but not in the BPD cohort. **d** Contrasting disgust vs. neutral we find a habituation effect for both HC and the BPD group. The asterisks denote significant differences between the first and the second run

We next tested for group differences specific to the self-conscious emotions shame and guilt. Contrasting shame and guilt scenarios versus disgust scenarios revealed stronger activations in the right amygdala in the BPD group compared to healthy controls (0.05 FWE corrected at the cluster level; Fig. 3a, Table 3e). We used cytoarchitectonic probability maps of the amygdala and the hippocampus [48] and the SPM Anatomy toolbox [49] to further specify the anatomical location of our findings. The cluster was mainly located in the superficial and laterobasal nuclear groups of the amygdala. The cluster maximum was assigned to the superficial nuclear group of the amygdala with a probability of 39%.The control group showed no amygdala reactivity for shame and guilt trials but increased activity for disgust scenarios, whereas the BPD group showed the opposite effect (Fig. 3b).

As the identical scenarios were presented in the three runs, we investigated how the amygdala reactivity evolved over time. Figure 3c, d shows the amygdala reactivity contrasting shame and guilt vs. neutral and disgust vs. neutral for each of the three runs separately. We found a significant decline of amygdala reactivity (between first and second run) in healthy controls when imagining shame and guilt scenarios (one-tailed paired *t*-test, *t*_21_ = 3.133, *p* = 0.003), and disgust scenarios (*t*_21_ = 1.972, *p* = 0.031). BPD patients, however, showed a significant decline for disgust scenarios (one-tailed paired *t*-test; *t*_18_ = 1.895, *p* = 0.037), but not for shame and guilt (*t*_18_ = 0.717, *p* = 0.241). The change in amygdala reactivity between the first and second run did however not reach significance when comparing BPD patients and the HC group (one-tailed two-sample *t*-test; *t*_39_ = − 1.563, *p* = 0.063). No further habituation effect for either of the two groups was found comparing the second and third run (all *p* > 0.05).

## Discussion

The aim of the current study was to investigate the neural basis of altered processing of self-conscious emotions in BPD by instructing participants to imagine fictitious scenarios with shame, guilt, disgust or neutral content. In terms of behavior, the main finding was in agreement with our hypothesis that women with BPD experience more intense feelings when being confronted with affective scenarios, especially higher levels of shame, guilt and fear. With regard to imaging results, we will discuss the main effect of emotional content and then elaborate on differences comparing women with BPD and healthy control subjects. Although we did not observe group differences specific to shame as compared to guilt, we found that women with BPD show generally enhanced amygdala reactivity when experiencing self-conscious emotions. The interaction of group with negative social (shame and guilt) and a negative non-social emotional condition (disgust) is of particular interest here as it allows to partial out effects related to self-relevance and social context rather than emotional valence.

### More intense emotional experience in women with BPD

The participants’ ratings demonstrated (i) that the experimental induction of target emotions was successful in both groups, (ii) that women diagnosed with BPD indicated more intense emotional experience, especially of shame and guilt and (iii) that the inner tension increased over time in participants of the BPD group, and decreased in the control group. As a limitation, one might argue that our focus on body shame and avoidance of possible triggers when selecting scenarios might have led participants to experience embarrassment rather than shame. Although rather similar to shame, embarrassment is less associated with morality and has been defined as “an aversive state of mortification, abashment, and chagrin that follows public social predicaments” [10, 50]. To answer this question, future studies should tap into other aspects of shame and ask for experience of embarrassment in addition to shame and guilt.

### Neural correlates of shame and guilt experience

Contrasting shame, guilt and disgust to neutral scenarios, we identified brain regions previously related to experiencing these emotions [32, 51], confirming the validity of our experimental manipulation. These regions are part of the networks implicated more generally in mentalizing and emotion understanding including middle and superior temporal gyrus, anterior insula and adjacent inferior frontal gyrus, the precuneus and the temporo-parietal junction [52–56]. Surprisingly, we did not find dorsomedial prefrontal areas and orbitofrontal cortex previously reported in research on self-conscious emotions [32]. Comparing shame and guilt scenarios, we found a significantly higher reactivity for shame scenarios in the posterior part of the medial temporal gyrus. This is in contrast to other studies comparing shame and guilt experience who reported either no shame-specific activation or in other areas [30, 32]. This speaks for the challenge and methodological heterogeneity in studying social emotions which was mentioned already in the introduction, which clearly limits the comparability across studies and thereby the conclusions we can derive about the neural networks specific to shame or guilt experience.

Contrasting emotional to neutral situations, we did not find significant between group effects when correcting for multiple testing. To explore the compatibility of our results with previous and future studies and meta-analyses, we also presented uncorrected results applying a voxel-wise *p*-threshold of *p *= 0.001 (Supplementary Table 4). Guilt scenarios resulted in a higher reactivity in BPD in the anterior insula (strongest effect), the angular gyrus, the precentral gyrus and the entorhinal area. In agreement with our finding, an increased activation of the anterior insula for BPD patients contrasting negative emotion and neutral trials was reported in a meta-analysis by Ruocco et al. [35]. The authors argue that an increased activation of the anterior insula might reflect a more intense subjective experience of negative emotion in BPD patients. Thus, whereas the pattern of results is similar to previous findings, we have to assert that based on more rigorous statistical testing, we do not find evidence for general differences in neural activity in women with BPD when experiencing socio-affective situations. The brain activation parameters did not correlate with BPD symptom severity or shame and guilt proneness which might most likely be due to small within-group variance in these measures, probably resulting from our extreme groups design. For future studies it might be interesting to also include a group with subclinical BPD symptoms.

As we will discuss in more detail in the next section, habituation effects play a crucial role in our experiment as the participants were confronted with an identical set of scenarios in each of the three runs. While this allows us to study differences in habituation between BPD patients and control participants, it might lead to decreased sensitivity to overall between-group differences and to the main effect of emotional content due to generally reduced effects in the second and third run.

### Reduced habituation to shame and guilt scenarios

Comparing women with BPD to healthy controls with respect to the self-conscious emotions guilt and shame, we found a significant interaction between emotional content and group in the right amygdala. The control group showed increased amygdala activity in disgust scenarios compared to shame and guilt scenarios. This is in accordance with previous studies in which the amygdala was not involved in processing shame and guilt [32], but in experiencing disgust [51]. The BPD group showed the opposite effect, namely increased amygdala activity for shame and guilt relative to disgust scenarios. In an activation-likelihood-estimation meta-analysis, Ruocco et al. [35] found reduced amygdala activation in BPD patients compared to HC contrasting negative emotion vs. neutral. In a review article, van Zutphen et al. [33] cited five fMRI studies finding increased amygdala activation in BPD processing emotional stimuli (faces and scenes). As mentioned already in the introduction, numerous explanations have been discussed to account for these inconsistencies including sample characteristics, task methodology, and psychological processes as habituation or dissociation [33, 35]. Addressing these criticisms, we included a non-social emotional condition (disgust), and successfully reduced the risk of dissociation, as neither post measurement questioning nor analysis of the distractor task suggested any dissociation in any of the subjects.

Our results suggest a complex and time-dependent involvement of the amygdala in the processing of emotional stimuli. We interpret the continuously increased amygdala activity in shame and guilt trials as a sign of decreased habituation in BPD, mirrored by a lack of decline in inner tension over the course of the BOLD measurement in this group. This effect was specific when experiencing self-conscious emotions. BPD patients showed the same effect of reduced amygdala activity over time (i.e., experimental runs) as HC when being confronted with disgust scenarios. This supports the notion of habituation differences in the work of van Zutphen et al. [33]. In support of Ruocco et al. [35], amygdala activity in BPD was not increased in the first run of shame and guilt vs. neutral trials. Our results are in line with fMRI results from Hazlett et al. [36]. They reported a potentiated amygdala response to repeatedly shown affective pictures in BPD. Patients exhibited normal amygdala activation during novel pictures but relatively increased amygdala activation during repeated pictures compared with HC. However, the authors used pictures from the IAPS [57], which only consider valence and arousal of the affective stimuli. Moreover, many of the IAPS pictures of negative valence show interpersonal threat or violence, which might trigger traumatic memories in persons with BPD more than in controls. With our study, we extend this work by showing reduced amygdala habituation to specifically self-conscious emotions and using more subtle and less salient stimuli.

From a psychotherapeutic point of view, the lack of amygdala habituation to shame and guilt in BPD patients might relate to the relatively slow improvement in emotional reactivity typically seen in therapy [58]. Clinical improvements following psychotherapy have been associated with a normalization of the amygdala response to visual stimuli with aversive affective content [59]. Whether this effect is specific to shame and guilt or extends to other aversive emotions (such as fear) remains to be studied. To date, established psychotherapies for BPD do not focus on the self-conscious emotions [24], but a small pilot study of dialectic behavioral therapy focusing on shame yielded promising results [60]. Our findings attest to the centrality of self-conscious emotions for BPD with more intensive subjective emotional experience and heightened neural response. However, we did not find any evidence for a specificity for shame as compared to guilt and the fMRI findings were overall rather small and clearly need replications with larger samples and other experimental approaches. Moreover, the number of trials for each condition and in each run was rather low as we aimed to use only well-defined scenarios meeting the above-mentioned criteria (see methods section) for our group comparison. While this reduced the heterogeneity across situations, the statistical power to examine condition differences and especially changes across experimental runs was reduced. This again calls for replication with other samples and other experimental approaches to substantiate our findings.

## Conclusions

The central finding in our study was the elevated amygdala reactivity in women with BPD when imagining shame- and guilt-related social scenarios. This was not due to increased activity per se in these scenarios. Rather, a diminished habituation to the presented stimuli across runs reflected an—on average—increased amygdala activity. This effect was specific to guilt and shame as BPD patients showed amygdala habituation comparable to healthy controls to a negative, but not self-conscious emotion (disgust). This finding helps to explain the inconsistencies between previous studies on the involvement of the amygdala in BPD as well as the typically slow progress in the psychotherapy of dysfunctional self-conscious emotions in this patient group.

## Electronic supplementary material

Below is the link to the electronic supplementary material.

Supplementary file 1 (DOCX 20 kb)
