# Supplementary material for: Neural basis of shame and guilt experience in women with borderline personality disorder
Source: Eur Arch Psychiatry Clin Neurosci. 2020 May 7;270(8):979–92. doi: 10.1007/s00406-020-01132-z (PMC7599192; doi:10.1007/s00406-020-01132-z)
Supplement: Supplementary file 1 — (DOCX 20 kb) [file 406_2020_1132_MOESM1_ESM.docx]

**Reduced amygdala habituation to shame and guilt scenarios in women with borderline personality disorder**

Martin Göttlich, Anna Lisa Westermair, Frederike Beyer, Marie Luise Bußmann, Ulrich Schweiger, Ulrike M. Krämer

**Supplementary Tables**

Supplementary Table 1. Previous and current mental health treatment

|  | Women with BPD  (N=19) | Healthy women  (N=22) |
| --- | --- | --- |
| Previous psychotherapy | 10.5 | 90.9 |
| None |  |  |
| For less than 1year | 21.1 | 9.1 |
| For one to 5years | 52.6 | 0.0 |
| For more than 5 years | 15.8 | 0.0 |
| Current pharmacotherapy | 10.5 | 59.1 |
| contraceptive |  |  |
| thyroid hormones | 21.1 | 9.1 |
| Proton pump inhibitor | 21.1 | 0.0 |
| Antihistamine | 15.8 | 0.0 |
| antihypertensive medication | 10.6 | 0.0 |
| Selective Serotonin RI | 15.8 | 0.0 |
| Serotonin-Norepinephrine RI | 47.4 | 0.0 |
| Tricyclic antidepressant | 10.5 | 0.0 |
| Antipsychotic* | 5.3 | 0.0 |
| Methylphenidate | 10.5 | 0.0 |
| Anti-epileptic drug* | 5.3 | 0.0 |
| *Notes*. Values indicate the percentage of participants having received/currently receiving the indicated mental health treatment. RI = reuptake inhibitor, * = for indications other than psychosis or epilepsy, respectively. | | |

Supplementary Table 2. Scenarios

| target emotion | original scenario | English translation |
| --- | --- | --- |
| shame | Sie gehen mit Freundinnen ins Kino und müssen wegen einer Blasenentzündung häufig auf die Toilette gehen. | You are at the cinema with some friends and need to go to the bathroom repeatedly due to a bladder infection. |
|  | In einer großen Gruppe sagt Ihnen jemand laut, dass Sie etwas Grünes zwischen den Zähnen haben. | In a group of people someone says loudly that you have something green between your teeth. |
|  | Auf einer Party bemerken Sie erst durch die Blicke der Anderen, dass Ihre Hose am Po ein großes Loch hat. | At a party, other people looking at your behind make you realize the large hole in your trouser. |
| \| guilt \| \| --- \| | Sie laufen hastig um die Ecke und rempeln dabei ein kleines Kind an, das anfängt zu weinen. | You run hastily around the corner and in doing so run over a child who starts crying. |
|  | An einem kalten Wintertag trödeln Sie und deswegen muss Ihre Freundin draußen auf Sie warten. | On a cold winter day you dawdle so that your friend has to wait for you out in the cold. |
|  | Sie sind mit einer Freundin im Café und machen sich über eine Bekannte lustig, was diese zufällig mitanhört. | You are at a café with a friend making fun of another friend who coincidently listens in on your conversation. |
| disgust | Während Sie gemeinsam bei einer Freundin kochen, finden Sie schimmliges Brot im Küchenschrank. | While cooking at a friend’s house you find mildewed bread in her kitchen cupboard. |
|  | Sie haben eine Freundin zu einem Spaziergang eingeladen, und jetzt tritt sie dabei in Hundekot. | You have invited a friend to a walk, where she steps into dog dirt. |
|  | Eine Freundin serviert Ihnen stolz selbst gebackenen Nusskuchen, in dem Sie lange Haare finden. | A friend proudly serves you homemade nut cake in which you find some long hairs. |
| neutral | Sie gehen mit einer Freundin ins Café, und die Kellnerin kommt um ihre Bestellung aufzunehmen. | You are at a café with a friend and the waitress arrives to take your order. |
|  | Im Bus treffen Sie eine gute Bekannte und unterhalten sich kurz mit ihr über das schöne Wetter. | You meet a friend at the bus and chat with her about the nice weather. |
|  | Samstagabends sehen Sie sich mit ein paar Freundinnen auf der Couch einen Film an und trinken Tee. | At a Saturday night you watch a movie with some friends, sitting on the sofa and drinking tea. |

**Supplementary Table 3. Mean intensity ratings of specific emotions at the pilot study**

|  | | | | | | | | | |
| --- | --- | --- | --- | --- | --- | --- | --- | --- | --- |
|  | shame scenarios | | guilt scenarios | | disgust scenarios | |  | | |
|  | M | SE | M | SE | M | SE |  |  |  |
| shame | **4,27** | **0,52** | 3,67 | 0,49 | 1,53 | 0,22 |  |  |  |
| guilt | 1,63 | 0,31 | **6,07** | **0,46** | 1,13 | 0,13 |  |  |  |
| disgust | 1,40 | 0,21 | 1,00 | 0,00 | **5,07** | **0,53** |  |  |  |
| anger | 3,10 | 0,48 | 2,17 | 0,45 | 1,40 | 0,16 |  |  |  |
| fear | 1,63 | 0,27 | 1,73 | 0,31 | 1,30 | 0,24 |  |  |  |
| sadness | 1,70 | 0,20 | 1,93 | 0,28 | 1,17 | 0,11 |  |  |  |
| joy | 1,80 | 0,31 | 1,20 | 0,20 | 1,23 | 0,12 |  |  |  |
| surprise | 2,73 | 0,44 | 3,57 | 0,56 | 2,87 | 0,43 |  |  |  |
| *Notes*. Target emotions are bolded. M = mean, SE = standard error. n = 10 healthy women. | | | | | | |  |  |  |

Supplementary Table 4. Imaging results: Between-group differences (uncorrected).

| *Brain region* | *Hem.* | *p (FWE)*  *cluster* | *Cluster*  *size* | *p (FWE)*  *peak* | *T*  *peak* | *MNI*  *coord.[mm]* |
| --- | --- | --- | --- | --- | --- | --- |
| 1. **BPD>HC (guilt vs. neutral)** | | | | | | |
| Anterior insula/medial orbitofrontal gyrus | L | 0.308 | 71 | 0.048 | 5.23 | -20,26,-12 |
| Anterior cingulate cortex/superior frontal gyrus (medial) | L | 0.713 | 26 | 0.678 | 3.96 | -10,28,30 |
|  |  |  |  | 0.892 | 3.66 | -17,30,28 |
|  |  |  |  | 0.911 | 3.62 | -27,28,28 |
| Precentral gyrus | L | 0.749 | 23 | 0.776 | 3.84 | -30,-24,70 |
|  |  |  |  | 0.967 | 3.45 | -37,-32,68 |
| Entorhinal area | R | 0.898 | 10 | 0.798 | 3.81 | 23,3,-30 |
|  |  |  |  | 0.928 | 3.58 | 28,-4,-35 |
| Angular gyrus | L | 0.877 | 12 | 0.844 | 3.74 | -50,-72,28 |
|  |  |  |  |  |  |  |
|  |  |  |  |  |  |  |
| 1. **BPD<HC (disgust vs. neutral)** | | | | | | |
|  |  |  |  |  |  |  |
| Inferior frontal gyrus (opercular part) | R | 0.772 | 21 | 0.702 | 3.93 | 53,10,18 |
| Supramarginal gyrus | R | 0.678 | 29 | 0.837 | 3.75 | 50,-24,38 |
| Precentral gyrus | R | 0.887 | 11 | 0.878 | 3.68 | 58,6,35 |
| *Notes*. Between-group effects for guilt vs. neutral (A) and disgust vs. neutral (B) scenarios (p<0.001 uncorrected; cluster size k>=10). The table shows three local maxima more than 8.0 mm apart. | | | | | | |
